# MicroRNA-378 regulates adipogenic differentiation in bovine intramuscular preadipocytes by targeting *CaMKK2*

**DOI:** 10.1080/21623945.2021.1982526

**Published:** 2021-10-24

**Authors:** Dongwei Li, Heng Wang, Yongmin Li, Changqing Qu, Yunhai Zhang, Hongyu Liu, Xiaorong Zhang

**Affiliations:** a College of Animal Science and Technology, Anhui Agricultural University, Hefei China; b Conservation Biology Research Center, School of Biology and Food Engineering, Fuyang Normal University, Fuyang, China; c Anhui Provincial Engineering Technology Research Center of Anti-aging Chinese Herbal Medicine, Fuyang Normal University, Fuyang, China

**Keywords:** Microrna-378, *Camkk2*, adipogenic differentiation, bovine, intramuscular preadipocytes

## Abstract

Intramuscular fat, as one of the most important palatability attribute of beef carcase, is the primary determinant of beef quality. The research of adipogenesis mechanism would provide new insight into intramuscular fatty deposition. Here, the role of microRNA-378 was investigated during bovine adipogenic differentiation. It was revealed that miR-378 expression exists variably in bovine major tissue and organs by RT-qPCR. It was predicted that miR-378 targets CaMKK2, as an AMPKα kinase, by DIANA Tools. For better research, primary preadipocytes with stable transfection for up-/down-regulated expression of miR-378 were constructed by lentiviral vectors with GFP gene. The analyses of qPCR showed that *PPARγ* and *adiponectin* mRNA levels increased, but *C/EBPβ, pref-1* and *CaMKK2* mRNA levels decreased during adipogenic differentiation. When miR-378 was overexpressed, preadipocytes proliferation became slower, there are more cellular lipid droplets, and *PPARγ* and *C/EBPβ* mRNA levels were higher, but *pref-1, adiponectin* and *CaMKK2* were lower than control groups. Luciferase assay and western blot analysis validated that miR-378 binds the nucleotide sites of the 3′- untranslated region of *CaMKK2*, which inhibits the mRNA and protein expression of *CaMKK2*. These findings suggest that miR-378 promotes adipogenic differentiation in bovine intramuscular preadipocytes by targeting *CaMKK2* via AMPK signalling pathway.

## Introduction

With the improvement of human living standards in modern society, the quality of food is paid more and more attention, people′s ideas from how to eat to how to eat well and healthily transition. People usually avoid eating too much fat. Fat (adipose tissue) originates from the propagation and differentiation of resident precursors represented by preadipocytes (hyperplasia) and the increase in size of adipocytes (hypertrophy), which plays important roles in energy balance as the fat storage depot for excess calories, some hormones and cytokines secretion, and the pathogenesis of many diseases such as obesity-related disorders [1]. However, beef fat is increasingly being valued by breeders for the flavour as well as its high protein value as a food source. During the growth and development of beef cattle, sufficient intramuscular adipose may improve beef tenderness and palatability, which is of huge interest for breeders and meat-eaters. Nevertheless, only improving the intramuscular adipose is very difficult. Adipogenesis is a complex biological process regulated by a cascade of transcription factors, including the proliferation, differentiation and maturation of preadipocytes. In the past decade, the research on adipogenesis is focused on human and experimental models rather than livestock, especially beef cattle. Currently, the mechanism of adipogenesis in beef intramuscular fat has not been clearly elucidated.

MicroRNAs (miRANs) are endogenous, single-stranded, noncoding RNA molecules, consisting of 20–24 nucleotides approximately, which primarily regulate mRNA transcription by targeting 3′-untranslated region (3′-UTR) of genes [2]. Now the special roles of many miRNAs in biology, pathology and others are unclear, functional characterizations of which suggest that they are unable to be ignored in processes of animal growth, development and physiology [3–5]. MiRNAs regulate the adipogenesis involving signal pathways, including PPAR, MAPK, cAMP/PKA and Wnt, or transcription factors such as *PPARγ* and *C/EBPs* and other series of genes [6,7]. For examples: miR-33a, miR-145, miR-150, miR-376a and miR-2400 could regulate bovine adipogenesis by targeting different genes of interest or changing the activity of special signalling pathways [8–12]. It was reported that miR-378/378* controls mitochondrial metabolism, systemic energy homoeostasis, including the size increase of lipid droplets and triacylglycerol generation against obesity [13–15]. MiR-378 and its host gene peroxisome proliferative activated receptor gamma coactivator 1β (*PPARGC1β, PGC1β*) were responsive to the PPARγ ligand rosiglitazone, allowing both to function synergistically in the regulation of lipid metabolism [16]. In previous researches, miR-378 regulates bovine adipogenesis by targeting *PPARγ*, mitogen-activated protein kinase 1, E2F transcription factor 2 and Ras-related nuclear-binding protein 10 [17,18]. However, the role of miR-378 in the differentiation of bovine intramuscular adipocytes remains unclear. In current study, we examined the expression of miR-378 in bovine intramuscular preadipocytes, majority of organs and tissue and investigated the effects of miR-378 on intramuscular preadipocytes propagation, differentiation and the underlying regulatory pathway, so as to provide references for improvement of beef quality.

## Material and methods

### Animal

Two newborn healthy bullocks were selected from the Easter Anhui Cattle Conservation Farm (Fengyang, Anhui Province, China). The longissimus thoracis muscles were collected under sterile conditions from the bullocks after they were euthanized as painless as possible according to the guidelines of livestock and poultry slaughtering procedure (GB/T 19477–2018).

### Preadipocytes isolation and cell culture

Primary preadipocytes were pooled from the longissimus dorsi muscles of several local cattle under good conditions. After samples were transported to the lab as soon as possible, the primary preadipocytes were cultured in the DMEM/F12 medium (Hyclone, USA) containing 10% foetal bovine serum (Hyclone, USA) in the 5% CO_2_ humidified atmosphere at 37.5°C. Once growth of cells was in close proximity to 100% confluence, the mixture of 0.2 μM insulin, 0.1 μM 3-isobutyl-1-methylxanthine, 0.1 μM dexamethasone and 0.1 μM rosiglitazone (Sigma, USA) was decanted into the DMEM/F12 to induce preadipocyte differentiation.

### Oil Red O staining extraction assay

The adipocytes were stained with Oil Red O. In brief, after the medium was removed, the adipocytes were rinsed twice gently with phosphate buffer solution and subsequently fixed with 4% paraformaldehyde solution at approximately 25°C for 30 minutes. Then, these cells were washed twice carefully with phosphate buffer solution and stained with Oil Red O working solution (3:2 dilution in distilled water and filtered with filter paper) for 30 minutes. The cells were then rinsed three times with phosphate buffer solution. The stained adipocytes were visualized under a light microscope, and the triglyceride content of the adipocytes was evaluated after isopropanol extraction by a UV-VIS spectrophotometer at 490 nm.

### Lentiviral vector construction

The miR-378 (MIMAT0009305) sequence was acquired from miRBase. The sequences of cDNA containing the miR-378 precursor, antagomir or negative base pairs were synthesized according to the desired gene sequence (Supplementary Fig. S1, Supplementary Table S1) and then the cDNA sequences were introduced into pcDNA™ 6.2-GW/EmGFP miR expression vectors after the oligonucleotide chains were annealed to generate recombinant clones by the BLOCK-iT™ Pol II miR RNAi Expression Vector Kit with EmGFP (Invitrogen, USA). The recombinant vectors were transformed into DH5α cells and verified by sequencing. The purified recombinant vectors were linearized with EagI (NEB, USA). Lentiviral vectors were constructed with the BP and LR recombination reaction system containing pDONR221 and the pLenti6.3/V5-DEST vector (Invitrogen, USA). Then, 293 T cells were simultaneously transfected by the lentiviral vectors and the packaging vectors with Lipofectamine 2000 (Sigma, USA). The viral titre was measured by the well dilution method.

### Cell transfection

Preadipocytes were seeded in 96-well culture plates. When these cells grew by 60–70% into the optimal infection stage, the preadipocytes were infected with 2 µl of the miR-378 recombinant lentivirus (titre: 1 × 10^8^ TU/mL, MOI = 100) as the miR-378 overexpression group. The preadipocytes were infected with 2 µl of anti-miR-378 recombinant lentivirus (titre: 1 × 10^8^ TU/mL, MOI = 100) as the anti-miR-378 group. And the preadipocytes were infected with 2 µl of the empty lentiviral vector (titre: 1 × 10^8^ TU/mL, MOI = 100) as the empty vector group. The preadipocytes were not infected with lentivirus as the control group of untreated cells. The stabilized infected monoclonal cell lines with lentivirus were acquired by blasticidin S selection (optimal concentration: 5.0 µg/mL). The miR-378 mRNA expression level of monoclonal cells in each experimental group was measured by RT-qPCR.

### PCR method

The genes mRNA expression levels were determined using SYBR-Green-based qPCR and the High Capacity cDNA Reverse Transcription Kit (Applied Biosystems) with the ABI StepOne Plus system (Applied Biosystems). The qPCR included an initial incubation at 95°C for 30 seconds, followed by 40 cycles of 95°C for 5 seconds and 60°C for 31 seconds. The relative miRNA expression levels were calculated using the delta delta Ct method with endogenous control of U6 snRNA. The reverse primer of miR-378 was Uni-miR qPCR primer (TaKaRa, China). Information regarding the other primer pairs used for qPCR is provided in Supplementary Table S2. The related quality tests for RNA extraction, reverse transcription, amplification efficiency of qPCR were performed as described in our previous publication [19].

### Cells proliferation and migration

Preadipocytes proliferation was detected using the cell counting technique. These cells were seeded in 6-well culture dishes and counted every other day until they reached the stationary phase. The cell growth curves were plotted with arithmetic averages, and the cell doubling time (DT) was calculated with the formula DT = ΔT×lg2/(lgNt_lgN_0_). Among of the formula, T is the cell culture time, Nt is the number of cells *in vitro* culture at time T, and N_0_ is the initial number of cells in vitro culture.

After the preadipocytes were cultured for 24 hours under the standard conditions, the cell culture medium was replaced with serum-free medium. The serum-free medium including 50,000 cells was added into a single transwell, which was placed in one well of the 24-well plate filled with medium containing 10% FBS. After normal culturing for 8 hours, the cells on the bottoms of the trans-/intra-wells of the 24-well plates were observed and counted under an inverted microscope, respectively.

### Dual luciferase reporter assay

Calcium/calmodulin-dependent protein kinase kinase 2 (*CaMKK2*) was predicted to be an important target gene of miR-378 by DIANA Tools. The 3′-UTR sequence and coding sequence of CaMKK2 were obtained from online databases of the National Center for Biotechnology Information, and the fragments were amplified by qPCR with the following primer pairs: 5′-ATGAGCTCGGTGTATGATCAGTTAG-3′ (forward) and 5′-ATAAGCTTTTCACAGTTACCCACACGG-3′ (reverse). More than 200 base pairs of the flanking regions of the binding sites were amplified inside the seed sequences predicted by bioinformatics, and the Hind III and Sac I restriction sites were both included in the amplified regions. The primer pairs for amplification of the mutant fragments by qPCR were 5′-GTCCCCTTTTGTAGCCAGCATTAAATAAA GAAAAAAGTTTACG-3′ (forward) and 5′-CGTAAACTTTTTTCTTTATTTAATGC TGGCTACAAAAGGGGAC-3′ (reverse). The qPCR conditions included an initial incubation at 94°C for 2 minutes, 35 cycles of 94°C for 30 seconds, 55°C for 30 seconds, and 68°C for 2 minutes, and 68°C for 8 minutes. Subsequently, 293 T cells were plated at 10^5^ cells per well before transduction. Then, the cells were co-transfected with miR-378 mimics, inhibitors or negative control oligonucleotides., which were examined with the Dual-Glo luciferase assay system (Promega, USA) after 24 hours.

### Western blot

Preadipocytes were collected when which grew by roughly 1 × 10^7^ in vitro culture and washed with phosphate buffer solution. Then, the cells were added into the total protein extract, which contained less than 1 ml of protease inhibitor (SinoGene, Beijing). The protein concentration in the lysate was calculated by the Bradford protein assay reagent (SinoGene, Beijing). The protein products were separated by SDS-PAGE and then transferred to PVDF membranes. The membranes were incubated with the following primary antibodies: anti-CaMKK2 and anti-β-actin (Abcom, England). Subsequently, the membranes were immunoblotted with the secondary goat anti-rabbit antibody after incubating overnight with primary antibodies. The immunoreactivity was visualized by enhanced chemiluminescence (ECL, Engreen).

### Statistical analysis

All data are presented as the means ± standard errors. One-way analysis of variance (ANOVA) was used to compare both groups, and SPSS software was used for multiple comparisons in the tests. *P*-values less than 0.05 and 0.01 were regarded as significant and highly significant, respectively.

## Results

### Expression of miR-378 in bovine major tissue, organs and intramuscular preadipocytes

**Figure 1.**
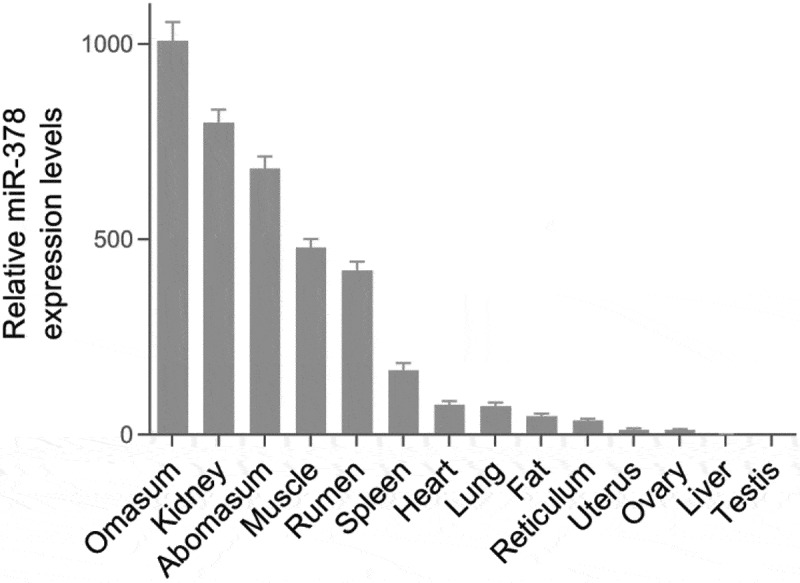
Relative expression levels of miR-378 in major bovine organs and tissue

**Figure 2.**
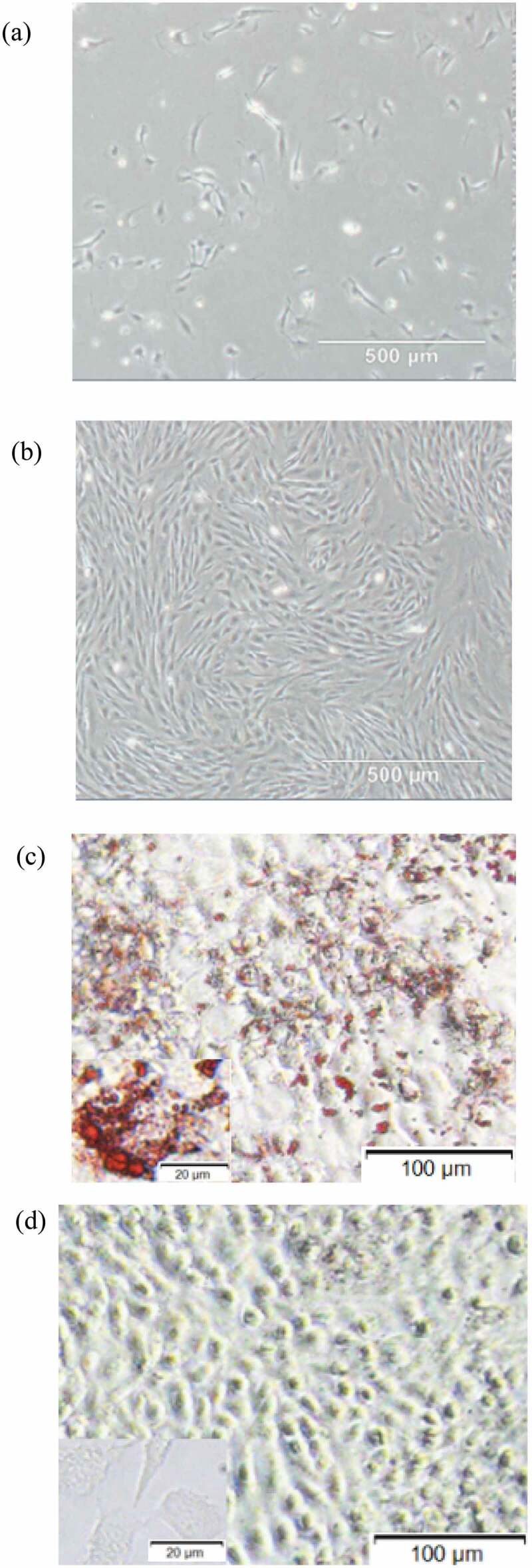
(a) Morphology of preadipocytes after 2 days of adipogenic differentiation. (b) Morphology of preadipocytes after 7 days of adipogenic differentiation. (c) Oil Red O staining of adipocytes after 7 days of adipogenic differentiation. (d) Oil Red O staining of adipocytes after 7 days culture without adipogenic differentiation

**Figure 3.**
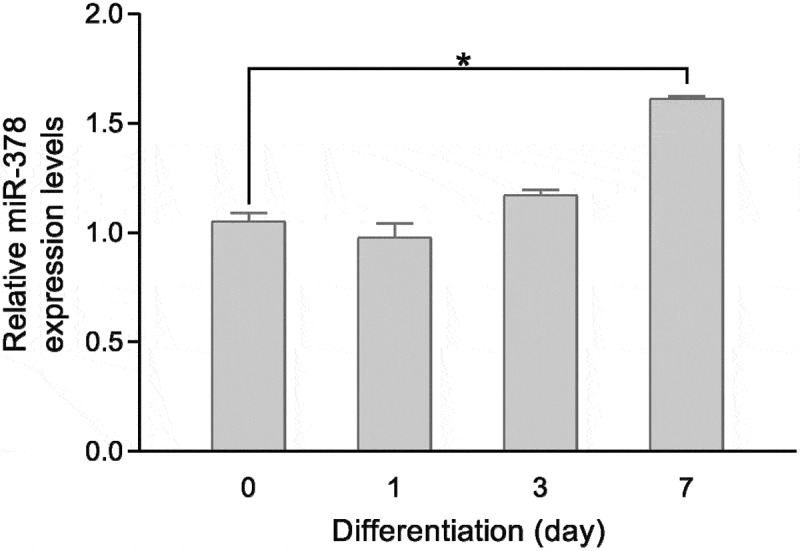
Relative expression levels of miR-378 during adipogenic differentiation. All data are presented as the means ± SE, * *P* < 0.05

The expression levels of miR-378 were examined in bovine major organs, tissue and preadipocytes by RT-qPCR. It is found that miR-378 expression levels were significantly different in the stomach, kidney, heart, liver, muscle, fat, uterus, ovary, and testis (Figure 1). Fibroblast-like bovine intramuscular preadipocytes were passaged and then induced into adipogenic differentiation before stopping growing with contact inhibition. The adipogenic differentiation of bovine intramuscular preadipocytes was examined with Oil Red O staining (Figure 2(a-d)). The expression level of miR-378 significantly increased after 7 days of adipogenic differentiation (Figure 3).

### Effect of miR-378 on proliferation, migration and adipogenic differentiation of bovine intramuscular monoclonal preadipocytes with stable transfection

**Figure 4.**
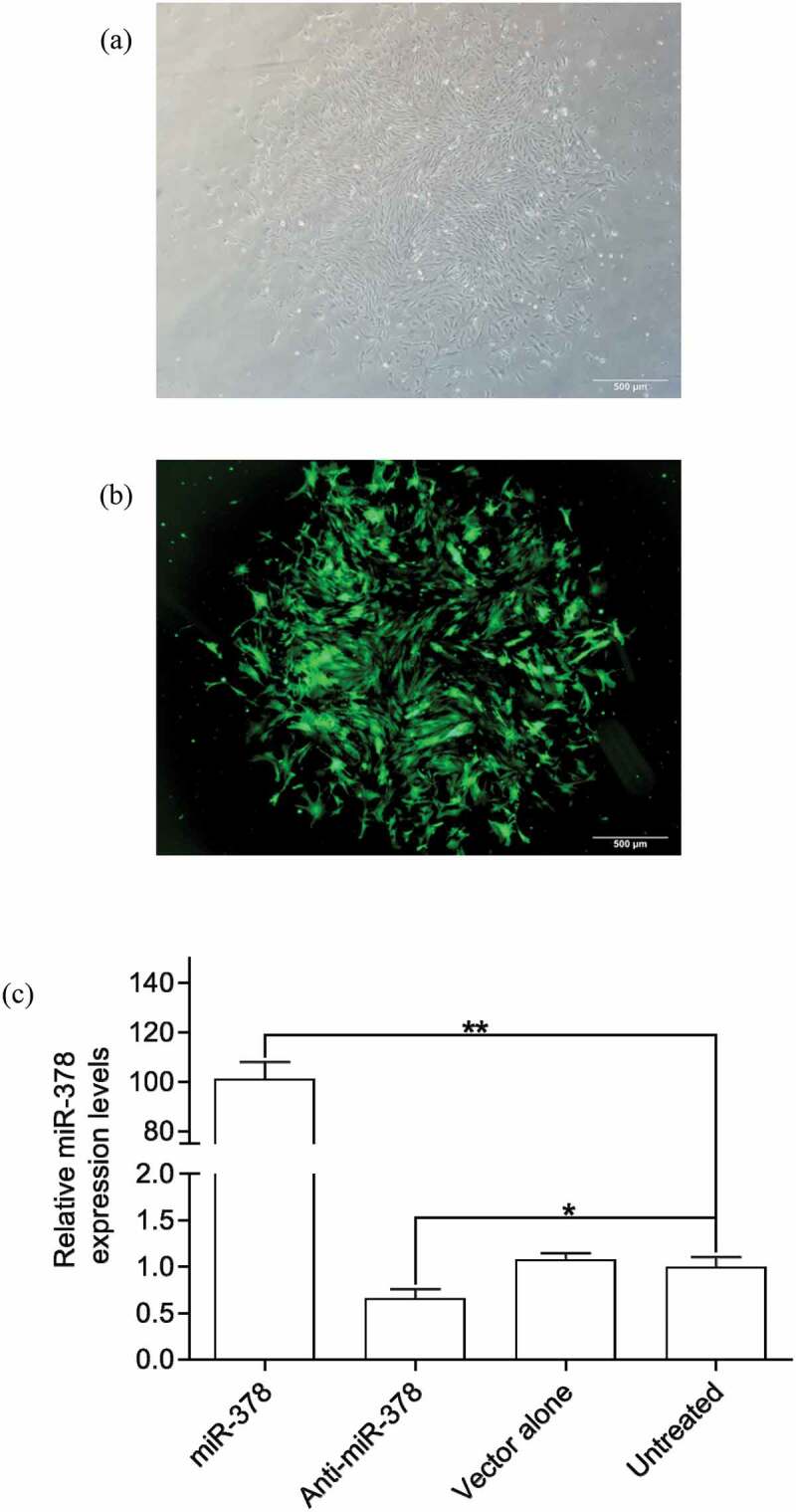
Relative expression levels of miR-378 in respective monoclonal preadipocytes. (a) Monoclonal preadipocytes in the neutral visual field; (b) Monoclonal preadipocytes in green fluorescent visual field. (c) Relative expression levels of miR-378 in monoclonal preadipocytes by qPCR (miR-378: miR-378 expression up-regulated preadipocytes; Anti-miR-378: miR-378 expression down-regulated preadipocytes; Vector along: preadipocytes transfected by lentiviral empty vector; Untreated: untreated preadipocytes. The following symbols are the same with these). All data are presented as the means ± SEs, * *P* < 0.05, ** *P* < 0.01

**Figure 5.**
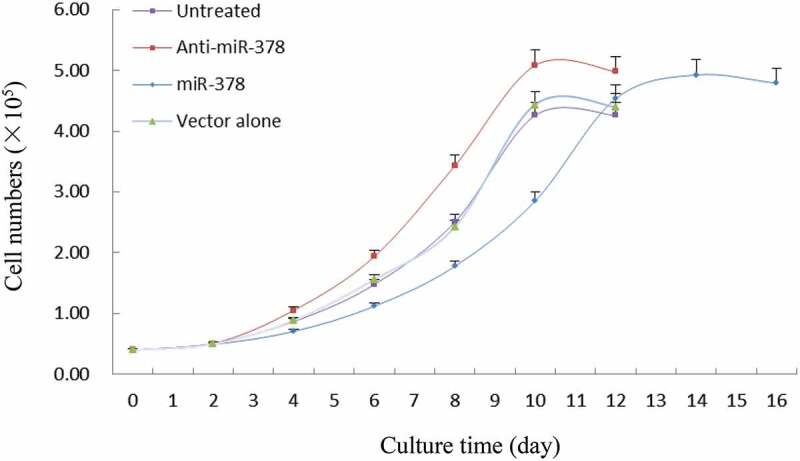
Proliferation curves of preadipocytes in groups. All data are presented as the means ± SE, * *P* < 0.05, ** *P* < 0.01

**Figure 6.**
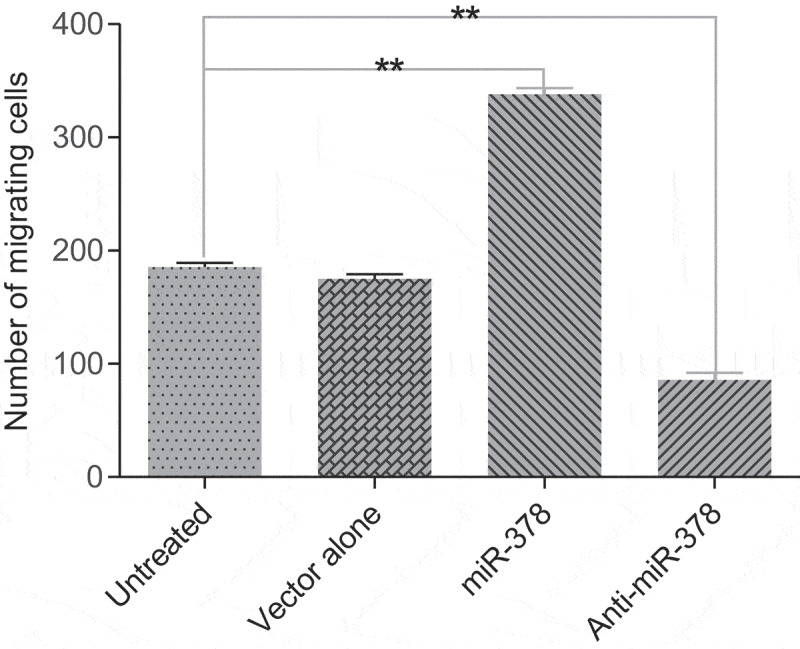
Effect of miR-378 expression level on the migration ability of preadipocytes. All data are presented as the means ± SE, * *P* < 0.05, ** *P* < 0.01

**Figure 7.**
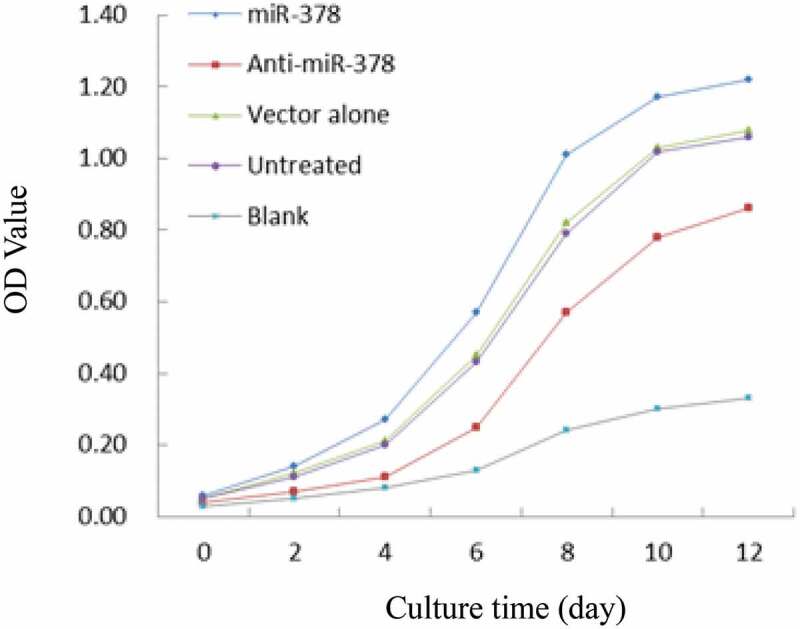
Cellular triglyceride content changes during bovine preadipocytes adipogenic differentiation (Blank: preadipocytes without induced adipogenic differentiation). All data are presented as the means

**Figure 8.**
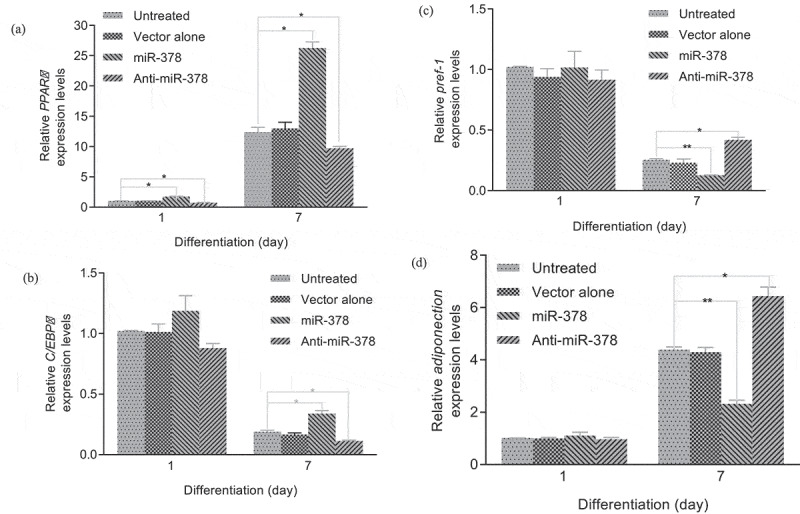
Relative expression levels of adipogenic genes during adipogenic differentiation. (a) Relative expression level of *PPARγ* mRNA. (b) Relative expression level of *C/EBPβ* mRNA. (c) Relative expression level of *Pref-1* mRNA. (d) Relative expression level of *adiponectin* mRNA. All data are presented as the means ± SE, * *P* < 0.05, ** *P* < 0.01

Primary preadipocyte lines with stable transfection for up-/down-regulated expression of miR-378 were constructed by lentiviral vectors with GFP gene, in order to investigate miR-378 effects on preadipocyte adipogenic differentiation. The preliminary test of lentiviral transduction demonstrated high efficiency (Supplementary Figure S2). The monoclonal preadipocytes, which were obtained via antibiotic-resistance-based selection after optimization of multiplicity of infection (MOI) by cloning cylinders, were found with stable over-/low-expression of miR-378 in an isolated culture system (Supplementary Figure S3, Supplementary Figure S4, Figure 4(a-c)). The results of the cell proliferation assays showed that the preadipocytes proliferation rate increased obviously, when miR-378 expression was inhibited. And the opposite happened, when miR-378 was overexpressed (Figure 5). The cell migration assay showed that the movement velocity of preadipocytes was increased, when miR-378 was overexpressed. When the expression of miR-378 was inhibited, the movement velocity was decreased (Figure 6; Supplementary Figure S6). Oil Red staining indicated that the triglyceride accumulation of adipocytes was decreased, when miR-378 expression was inhibited. But miR-378 overexpression raised the triglyceride level of adipocytes (Figure 7). When miR-378 was overexpressed, the mRNA levels of *PPARγ, C/EBPβ* and *adiponectin* were significantly increased, and the mRNA level of *Pref-1* was decreased after 7 days of adipogenic differentiation. When miR-378 expression was inhibited, those demonstrations would be inversed (Figure 8(a-d)). The results suggested that miR-378 attenuates preadipocytes propagation, promote preadipocytes migration and adipogenic differentiation.

### MiR-378 regulate adipogenic differentiation of bovine intramuscular preadipocytes by targeting 3′-UTR of CaMKK2

**Figure 9.**
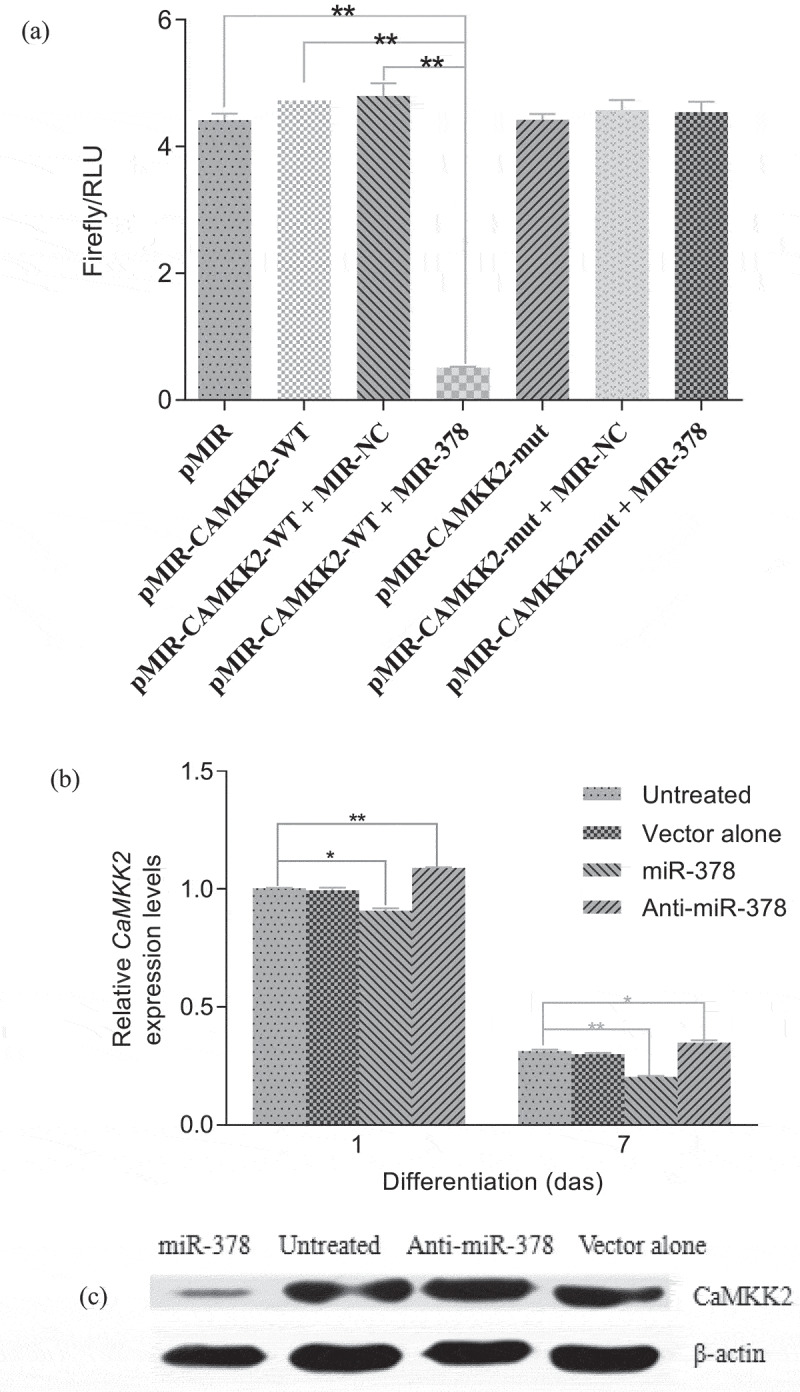
MiR-378 facilitates adipogenic differentiation in bovine intramuscular preadipocytes by targeting *CaMKK2*. (a) miR-378 targets the 3′-UTR of *CaMKK2*, which is verified by the dual luciferase Reporter Assay. (b) Relative expression levels of *CaMKK2* mRNA after 1 day and 7 days of adipogenic differentiation. (c) Effect of miR-378 expression on *CaMKK2* protein translation by western blot. All data are presented as the means ± SE, * *P* < 0.05, ** *P* < 0.01

*CaMKK2*, which is bound up with the AMPK physiologically in mammalian cells, was predicted to be an important target gene of miR-378 by DIANA Tools (Supplementary Figure S5). To verify that *CaMKK2* is targeted by miR-378, dual luciferase reporter assay was performed. Sequence alignment of the 3′-UTR of *CaMMK2* and miR-378 mature sequence was illustrated with the bioinformatics algorithm. The seed sequence in the 3′-UTR of *CaMMK2* was mutated to detect the interaction of miR-378 and the 3′-UTR of *CaMMK2*, and then it was found that the relative luciferase activity was not influenced by miR-378 overexpression. However, the relative luciferase activity was strongly reduced when overexpressed miR-378 acted on the normal 3′-UTR of *CaMMK2* (Figure 9(a)). The transcription and translation levels of *CaMKK2* were examined after 1 day of adipogenic differentiation. When miR-378 was overexpressed, *CaMKK2* mRNA and protein expression levels of preadipocytes were significantly lower than those of the untreated or blank-load-transfected preadipocytes by RT-qPCR and western blot analysis (Figure 9(b-c)).

## Discussion

Adipose cells are not only storage depots of triacylglycerol, but also secretors of adipokines exerting pleiotropic function under the impact of various cytokines, hormones and nutrients [20]. And intramuscular fatty deposition in livestock is crucial for meat quality, which would affect the taste and tenderness of meat. The adipogenesis of livestock includes a series of complex molecular events, and details of which remain unknown, such as the roles of miRNAs. Here, we investigated the contribution of miR-378 to bovine intramuscular adipogenic differentiation.

Bovine preadipocytes were obtained by the monoclonal culture system, and then were successfully transfected by lentiviral vectors for miR-378 expression up-/down- regulation. Primary preadipocytes present essential physiological characteristics than the commercial 3T3-L1, NIH-3T3, etc. The effects of miR-378 on intramuscular preadipocytes proliferation, migration, triglyceride accumulation and the mRNA expressions of key adipogenic genes were consistent with previous reports [14,18]. The novel binding sites for miR-378 were predicted in the 3′-UTR of bovine *CaMKK2* by the bioinformatics algorithm, and then this was verified by the dual luciferase reporter assay and western blot. The expression of miR-378 was correlated inversely with luciferase activity in bovine intramuscular preadipocytes, as well as the mRNA and protein expressions of *CaMKK2*.

*CaMKK2* exists in major internal organs, tissue and cells, involved in energy balance, glucose tolerance, inflammation, cancer and so on [21–25]. Nucleotides sequence analysis revealed species homogeneity in the protein-coding region, but heterogeneity in the 3′-UTR [20,26–29]. The functional mechanism of CaMKK2 is associated with AMPK. There is evidence that AMPK activation can inhibit preadipocyte differentiation [30,31]. As an alternative upstream kinase of AMPK, CaMKK2 phosphorylates AMPKα to reduce the mRNA levels of *Pref-1* and *Sox9* and then accelerate adipogenesis, which can be reversed by activation of 5-aminoimidazole-4-carboxamide ribonucleotide on AMPK [32–36]. A few researches are concerned with the correlation of miRNAs and *CaMKK2* in cancer [37,38]. So far, no such association has been reported in adipogenesis.

In conclusion, we have provided evidence that miR-378 regulates adipocytes differentiation in bovine intramuscular preadipocyte and propose that miR-378 plays a partial role by targeting the 3′-UTR of *CaMKK2 via* AMPK signalling pathway. Further studies would be enlightened on miR-378-*CaMKK2*-AMPK pathway. It suggests that miR-378 plays an important role in fat deposit associated with special implications for some energy metabolism disequilibrium. The findings contribute to a better understanding of miRNA action on adipogenesis and provide a significant reference for future breeding research on beef intramuscular fat.

## Supplementary Material

Supplemental Material

## Data Availability

The authors confirm that the data supporting the findings of this study are available within the article and its supplementary materials.

## References

[1] Ghaben AL, Scherer PE. Adipogenesis and metabolic health. Nat Rev Mol Cell Biol. 2019;20(4):242–258. 30610207 10.1038/s41580-018-0093-z

[2] Bartel DP. MicroRNAs: target recognition and regulatory functions. Cell. 2009;136(2):215–233. 19167326 10.1016/j.cell.2009.01.002 PMC3794896

[3] Ambros V. The functions of animal microRNAs. Nature. 2004;431(7006):350–355. 15372042 10.1038/nature02871

[4] Garcia AI, Miska EA. MicroRNA functions in animal development and human disease. Development. 2005;132(21):4653–4662. 16224045 10.1242/dev.02073

[5] Engin AB. MicroRNA and Adipogenesis. Adv Exp Med Biol. 2017;960:489–509. 28585213 10.1007/978-3-319-48382-5_21

[6] sá PM D, AJ R, Hang H, et al. Transcriptional regulation of adipogenesis. Compr Physiol. 2017;7:635–674. 28333384 10.1002/cphy.c160022

[7] Son YH, Ka S, Kim AY, et al. Regulation of adipocyte dierentiation via microRNAs. Endocrinol Metab. 2014;29(2):122–135. 10.3803/EnM.2014.29.2.122 PMC4091493 25031884

[8] Zhang WZ, Wang L, Raza SHA, et al. MiR-33a plays a crucial role in the proliferation of bovine preadipocytes. Adipocyte. 2021;10(1):189–200. 33840361 10.1080/21623945.2021.1908655 PMC8043176

[9] Wang L, Zhang S, Cheng G, et al. MiR-145 reduces the activity of PI3K/Akt and MAPK signaling pathways and inhibits adipogenesis in bovine preadipocytes. Genomics. 2020;112(4):2688–2694. 32135297 10.1016/j.ygeno.2020.02.020

[10] Chen XY, Raza SHA, Ma XH, et al. Bovine Pre-adipocyte Adipogenesis Is Regulated by bta-miR-150 Through mTOR Signaling. Front Genet. 2021;12:636550–636565. 33633792 10.3389/fgene.2021.636550 PMC7901978

[11] Chen XY, Raza SHA, Cheng G, et al. Bta-miR-376a Targeting KLF15 Interferes with Adipogenesis Signaling Pathway to Promote Dierentiation of Qinchuan Beef Cattle Preadipocytes. Animals (Basel). 2020;10(12):2362–2377. 10.3390/ani10122362 PMC7763857 33321855

[12] Wei Y, Cui YF, Tong HL, et al. MicroRNA-2400 promotes bovine preadipocyte proliferation. Biochem Biophys Res Commun. 2016;478:1054–1059. 27514450 10.1016/j.bbrc.2016.08.038

[13] Carrer M, Liu N, Grueter EC, et al. Control of mitochondrial metabolism and systemic energy homeostasis by microRNAs 378 and 378*. Proc Natl Acad Sci. 2012;109(38):15330–15335. 22949648 10.1073/pnas.1207605109 PMC3458360

[14] Gerin I, Bommer GT, McCoin SC, et al. Roles for miRNA-378/378* in adipocyte gene expression and lipogenesis. Am J Physiol Endocrinal Metab. 2010; 299: e198–206. 10.1152/ajpendo.00179.2010 PMC2928515 20484008

[15] Pan D, Mao CX, Quattrochi B, et al. MicroRNA-378 controls classical brown fat expansion to counteract obesity. Nat Commun. 2015;5(1):4725–4753. 10.1038/ncomms5725 PMC4167820 25145289

[16] John E, Wienecke-Baldacchino A, Liivrand M, et al. Dataset integration identifies transcriptional regulation of microRNA genes by PPARγ in differentiating mouse 3T3-L1 adipocytes. Nucleic Acids Res. 2012;40(10):4446–4460. 22319216 10.1093/nar/gks025 PMC3378868

[17] Jin W, Dodson VM, Moore SS, et al. Characterization of microRNA expression in bovine adipose tissues: a potential regulatory mechanism of subcutaneous adipose tissue development. BMC Mol Biol. 2010;11(1):29–37. 20423511 10.1186/1471-2199-11-29 PMC2874793

[18] Liu SY, Zhang YY, Gao Y, et al. MiR-378 Plays an Important Role in the Differentiation of Bovine Preadipocytes. Cell Physiol Biochem. 2015;36(4):1552–1562. 26159460 10.1159/000430318

[19] Li D, Liu HY, Li YS, et al. Identification of suitable endogenous control genes for quantitative RT-PCR analysis of miRNA in bovine solid tissues. Mol Biol Rep. 2014;41(10):6475–6480. 24973888 10.1007/s11033-014-3530-x

[20] Feve B. Adipogenesis: cellular and molecular aspects. Best Pract Res Clin Endocrinol Metab. 2005;19(4):483–499. 16311213 10.1016/j.beem.2005.07.007

[21] Anderson KA, Ribar JT, Lin F, et al. Hypothalamic CaMKK2 contributes to the regulation of energy balance. Cell Metab. 2008;7(5):377–388. 18460329 10.1016/j.cmet.2008.02.011

[22] Anderson KA, Lin F, Ribar JT, et al. Deletion of CaMKK2 from the liver lowers blood glucose and improves whole body glucose tolerance in the mouse. Mol Endocrinal. 2012;26(2):281–291. 10.1210/me.2011-1299 PMC3275166 22240810

[23] Racioppi L, Noeldnet KP, Lin F, et al. Calcium/calmodulin-dependent protein kinase kinase 2 regulates macrophage mediated inflammatory responses. J Biol Chem. 2012;287(14):11579–11591. 22334678 10.1074/jbc.M111.336032 PMC3322820

[24] Frigo DE, Howe KM, Wittmann MB, et al. CaM kinase kinase β-mediated activation of the growth regulatory kinase AMPK is required for androgen-dependent migration of prostate cancer cells. Cancer Res. 2011;71(2):528–537. 21098087 10.1158/0008-5472.CAN-10-2581 PMC3074523

[25] Colomer J, Means AR. Physiological roles of the Ca^2+^/CaM-dependent protein kinase cascade in health and disease. Subcell Biochem. 2007;45:169–214. 18193638 10.1007/978-1-4020-6191-2_7

[26] Vinet J, Carra S, Blom MCJ, et al. Cloning of mouse Ca^2+^ </sup>++/calmodulin-dependent protein kinase kinase beta (CaMKKβ) and characterization of CaMKKβ and CaMKKα distribution in the adult mouse brain. Brain Res Mol Brain Res. 2003;111(1–2):216–221. 12654522 10.1016/s0169-328x(02)00698-8

[27] Matsushita M, Nairn AC. Characterization of the mechanism of regulation of Ca^2+^/calmodulin-dependent protein kinase I by calmodulin and by Ca^2+^/calmodulin-dependent protein kinase kinase. J Biol Chem. 1998;273(34):21473–21481. 9705275 10.1074/jbc.273.34.21473

[28] Anderson AK, Means LR, Huang HQ, et al. Components of a calmodulin-dependent protein kinase cascade. Molecular cloning, functional characterization and cellular localization of Ca^2+^/calmodulin-dependent protein kinase kinase β. J Biol Chem. 1998;273(48):31880–31889. 9822657 10.1074/jbc.273.48.31880

[29] Hsu LS, Tsou PA, Chi WC, et al. Cloning, expression, and chromosomal localization of human Ca2^+^/calmodulin-dependent protein kinase kinase. J Biomed Sci. 1998;5:141–149. 9662074 10.1007/BF02258368

[30] Daval M, Foufelle F, Ferré P. Functions of AMP-activated protein kinase in adipose tissue. J Physiol. 2006;574(1):55–62. 16709632 10.1113/jphysiol.2006.111484 PMC1817807

[31] Habinowski SA, Witters LA. The effects of AICAR on adipocyte differentiation of 3T3-L1 cells. Biochem Biophys Res Commun. 2001;286(5):852–856. 11527376 10.1006/bbrc.2001.5484

[32] Lin F, Ribar TJ, Means RA. The Ca^2+^ </sup>++/Calmodulin-Dependent Protein Kinase Kinase, CaMKK^2+^+, Inhibits Preadipocyte Differentiation. Endocrinology. 2011;152(10):3668–3679. 21862616 10.1210/en.2011-1107 PMC3176646

[33] Fujiwara Y, Kawaguchi Y, Fujimoto T, et al. Differential AMP-activated protein kinase (AMPK) recognition mechanism of Ca^2+^/ Calmodulin-dependent protein kinase kinase isoforms. J Biol Chem. 2016;291(26):13802–13808. 27151216 10.1074/jbc.M116.727867 PMC4919462

[34] Fogarty S, Hawley AS, Green AK, et al. Calmodulin-dependent protein kinase kinase-beta activates AMPK without forming a stable complex: synergistic effects of Ca^2+^ and AMP. J Biol Chem. 2010;426(1):109–118. 10.1042/BJ20091372 PMC2830670 19958286

[35] Hawley SA, Pan AD, Mustard JK, et al. Calmodulin-dependent protein kinase kinase-β is an alternative upstream kinase for AMP-activated protein kinase. Cell Metab. 2005;2(1):9–19. 16054095 10.1016/j.cmet.2005.05.009

[36] Woods A, Dicherson K, Heath R, et al. Ca^2+^ </sup>++/calmodulin-dependent protein kinase kinase-β acts upstream of AMP-activated protein kinase in mammalian cells. Cell Metab. 2005;2(1):21–33. 16054096 10.1016/j.cmet.2005.06.005

[37] Fu H, He HC, Han ZD, et al. MicroRNA-224 and its target CAMKK2 synergistically influence tumor progression and patient prognosis in prostate cancer. Tumour Biol. 2015;36(3):1983–1991. 25394900 10.1007/s13277-014-2805-0

[38] Huang YK, Su YF, Lieu AS, et al. MiR-1271 regulates glioblastoma cell proliferation and invasion by directly targeting the CAMKK2 gene. Neurosci Lett. 2020;737:135289. 32791096 10.1016/j.neulet.2020.135289

