# Supplementary material for: MicroRNA-378 regulates adipogenic differentiation in bovine intramuscular preadipocytes by targeting CaMKK2
Source: Adipocyte. 2021 Oct 24;10(1):483–92. doi: 10.1080/21623945.2021.1982526 (PMC8547835; doi:10.1080/21623945.2021.1982526)
Supplement: Supplemental Material [file KADI_A_1982526_SM4950.docx]

MicroRNA-378 regulates adipogenic differentiation in bovine intramuscular preadipocytes by targeting *CaMKK2*

Dongwei Li^a, c*+^, Heng Wang^a+^, Yongmin Li^c^, Changqing Qu^b^, Yunhai Zhang^a^, Hongyu Liu^a*^, Xiaorong Zhang^a*^

^a^College of Animal Science and Technology, Anhui Agricultural University, Hefei China; ^b^Anhui Provincial Engineering Technology Research Center of Anti-aging Chinese Herbal Medicine, Fuyang Normal University, Fuyang, China; ^c^ Conservation Biology Research Center, School of Biology and Food Engineering, Fuyang Normal University, Fuyang, China


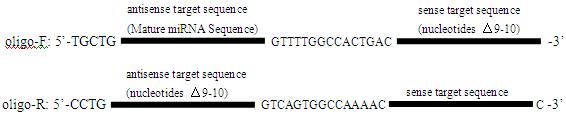


Supplementary Figure S1. cDNA oligonucleotide sequences based on miR-378

(b)

(a)


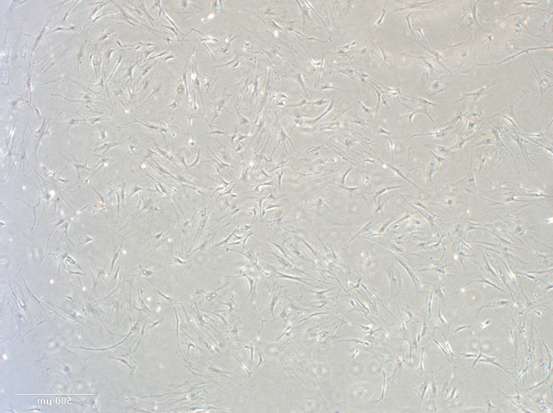

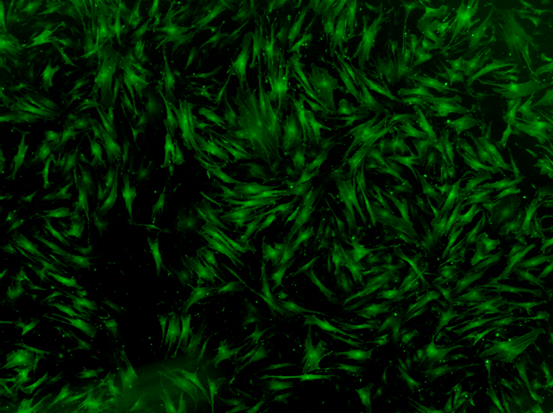


(d)

(c)


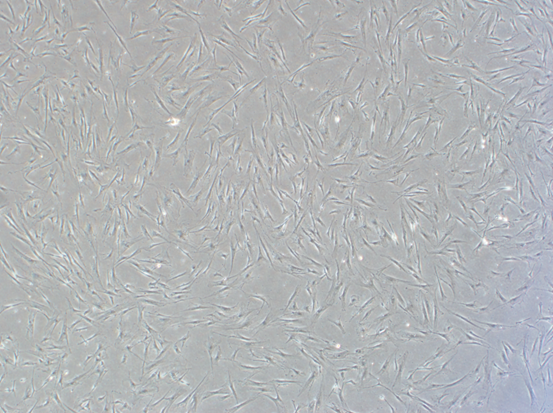

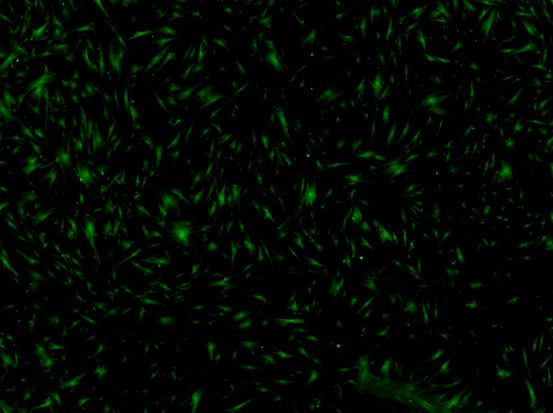


(e) (f)


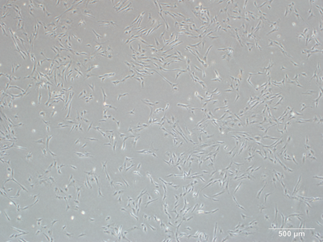

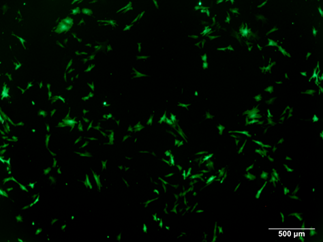


Supplementary Figure S2. Lentiviral vector transfection of bovine preadipocytes. (a),(b): Preadipocytes of miR-378 overexpression; (c),(d):Preadipocytes of miR-378 expression inhibition; (e), (f): Preadipocytes of transfected by lentiviral empty vector.

(a)

(b)


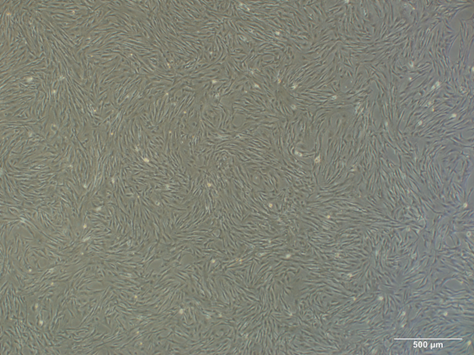

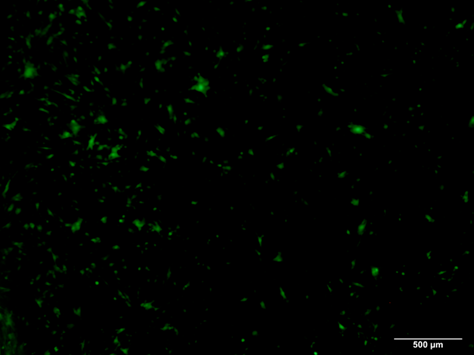


(d)

(c)


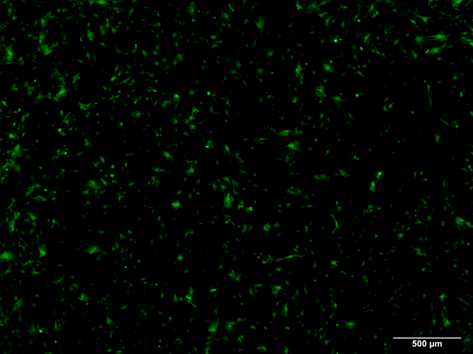

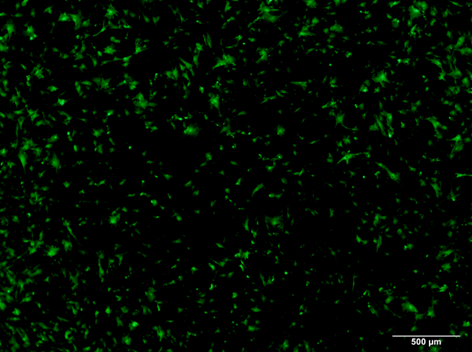


(f)

(e)


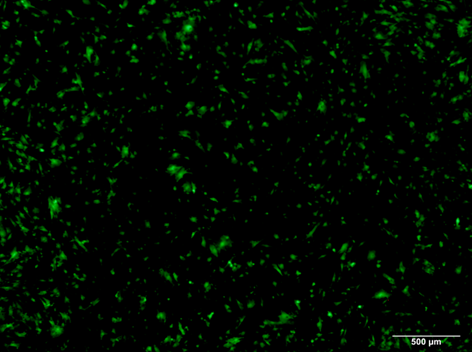

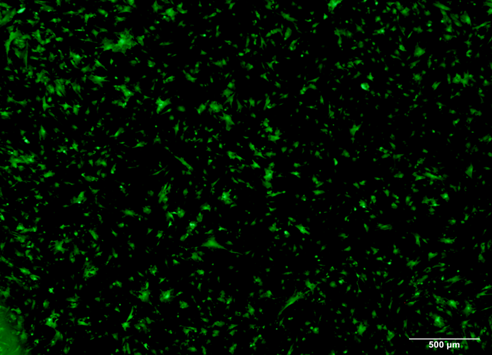


Supplementary Figure S3. MOI determination of bovine preadipocytes transfected with lentiviral vectors. (a): preadipocytes transfected at MOI 0; (b): preadipocytes transfected at MOI 20; (c): preadipocytes transfected at MOI 50; (d): preadipocytes transfected at MOI 100; (e): preadipocytes transfected at MOI 150; (f): preadipocytes transfected at MOI 200.

(a)

(b)


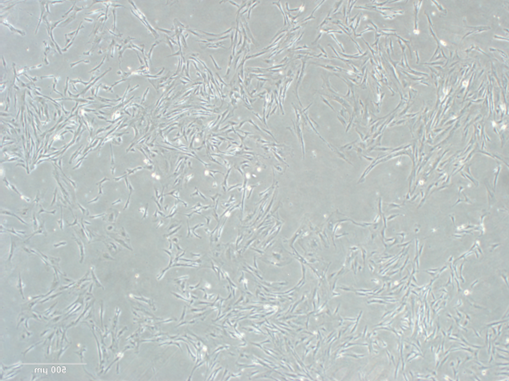

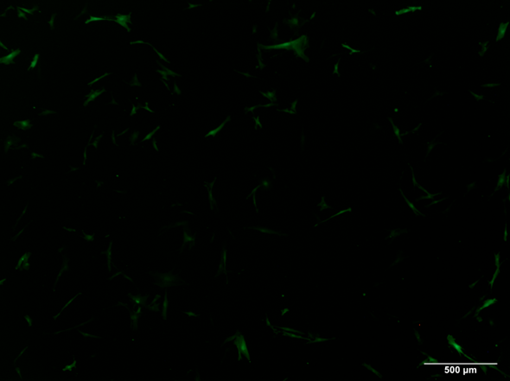


(d)

(c)


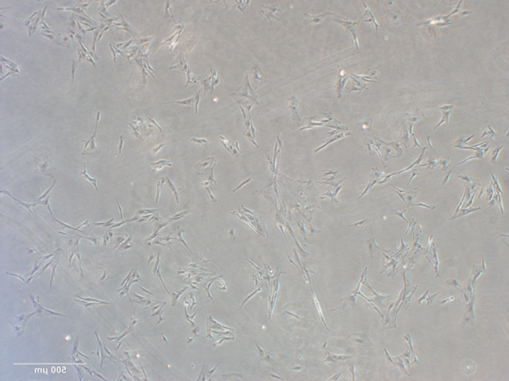

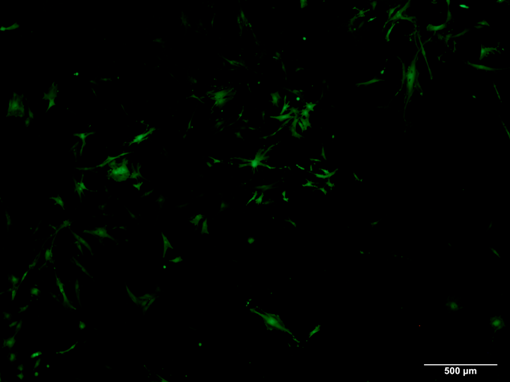


Supplementary Figure S4. Antibiotic-resistance-based cell selection after transfection by application of blasticidin S (a), (b): blasticidin S at a concentration of 2.0 μg/mL; (c), (d): blasticidin S at a concentration of 5.0 μg/mL.

Position on chromosome: 12:121677208-121677228

CaMKK2 3′ UTR 5′ AAGUUCCCCUUUGUAGCUAAAAGUCCAGA 3′

• •

miR-378 3′ CGGAAGAC UGAGGUUCAGGUCA 5′

Position on chromosome: 12:121676191-121676214

CaMKK2 3′ UTR 5′ GAGGUGCUGUUAUGAGCAGCUCU UCCAGU 3′

• •

miR-378 3′ CGGAAG ACU GAGGUUCAGGUCA 5′

Supplementary Figure S5. Conserved binding sites in CaMKK2 mRNA that aligned with miR-378 were predicted using DIANA Tools

.

Supplementary Table S1. Target cDNA oligonucleotide sequences

| Name | cDNA oligonucleotides sequences (5′-3′) |
| --- | --- |
| Oligonucleotides transformed to miR-378 precursor sequences | |
| 13MR0028-1F | TGCTGactggacttggagtcagaaggcGTTTTGGCCACTGACTGACgccttctgtccaagtccagt |
| 13MR0028-1R | CCTGactggacttggacagaaggcGTCAGTCAGTGGCCAAAACgccttctgactccaagtccagtC |
| Oligonucleotides transformed to miR-378 interference sequences | |
| 13MR0028-2F | TGCTGgccttctgactccaagtccagtGTTTTGGCCACTGACTGACactggactgagtcagaaggc |
| 13MR0028-2R | CCTGgccttctgactcagtccagtGTCAGTCAGTGGCCAAAACactggacttggagtcagaaggcC |
| Negative control | |
| Negative-F | tgctgAAATGTACTGCGCGTGGAGACGTTTTGGCCACTGACTGACGTCTCCACGCAGTACATTT |
| Negative-R | cctgAAATGTACTGCGTGGAGACGTCAGTCAGTGGCCAAAACGTCTCCACGCGCAGTACATTTc |

Supplementary Table S2. Primers for fluorescence-based quantitative RT-PCR

| Gene ID | Primer Sequence (5′-3′) | Length |
| --- | --- | --- |
| miR-378 | ACTGGACTTGGAGTCAGAAGG | 66 |
| U6 | CGCTTCACGAATTTGCGTGTCAT  GCTTCGGCAGCACATATACTAAAAT | 199 |
| CaMKK2 | GGTCCTTGTGTTGTTCTCT ACTCATCCATTCCTTCCTTC | 161 |
| PPARγ | AGTTTATTCCCACCTCCTCC | 317 |
|  | CACAAGATCAAGTCCACCCT |  |
| C/EBPβ | GCCAAGAAGACGGTGGACAAGC | 209 |
|  | TTGAACAAGTTCCGCAGGGTG |  |
| Pref-1 | TGCCTGAACCTCGATGACG | 194 |
|  | CAGAAGTTGCCCGAGAAGC |  |
| ADI | TCATTATGACGGCAGCAC | 194 |
|  | CCAGATGGAGGAGCACAG |  |


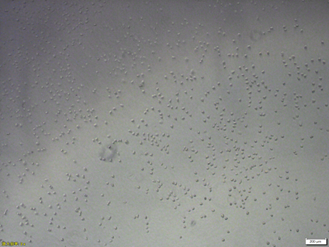

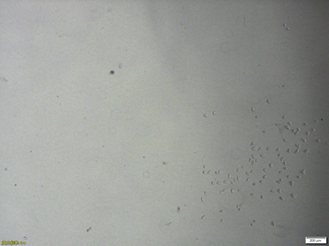


B

A


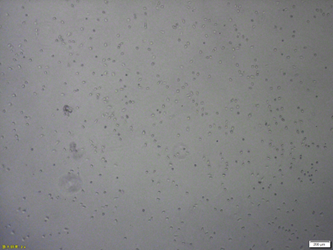

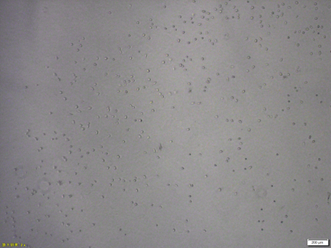


D

C

Supplementary Figure S6. The phenotype images of cell migration assay. A: the group of miR-378 overexpression. B: the group of miR-378 expression inhibition. C: the group transfected with empty vector alone. D: the untreated group.


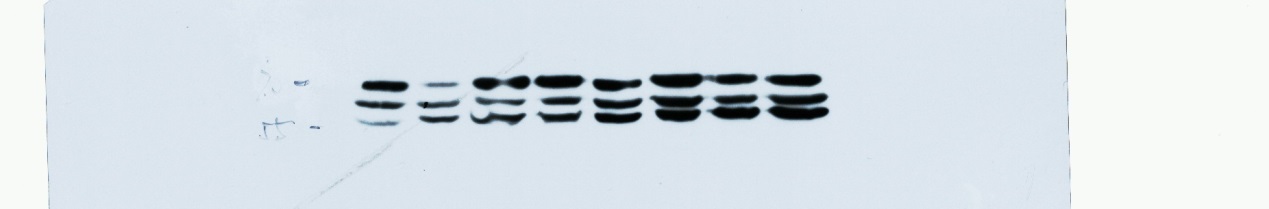


Supplementary Figure S7. Original CaMKK2 western blot

Notes: First row: lane 2, miR-378; lane 3, untreated; lane 4, anti-miR-378; lane 5, vector alone; others, unrelated.


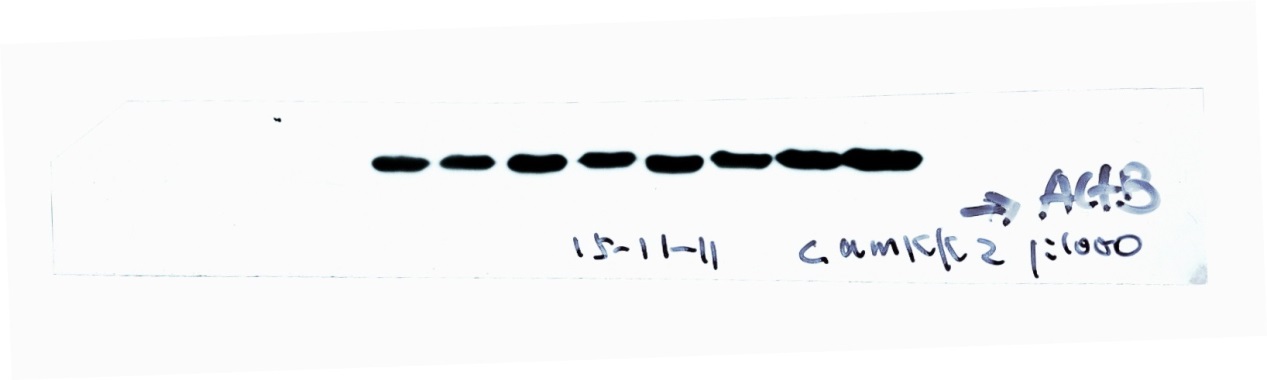


Supplementary Figure S8. Original β-actin western blot

Notes: Lane 2, miR-378; lane 3, untreated; lane 4, anti-miR-378; lane 5, vector alone; others, unrelated.
